# Supplementary material for: Genome-Wide Associations with Resistance to Bipolaris Leaf Spot (Bipolaris oryzae (Breda de Haan) Shoemaker) in a Northern Switchgrass Population (Panicum virgatum L.)
Source: Plants (Basel). 2022 May 20;11(10):1362. doi: 10.3390/plants11101362 (PMC9144872; doi:10.3390/plants11101362)
Supplement: Supplementary file 1 [file plants-11-01362-s001.zip › Songsomboon_Plant_supplement_figures_revision.pdf]

1

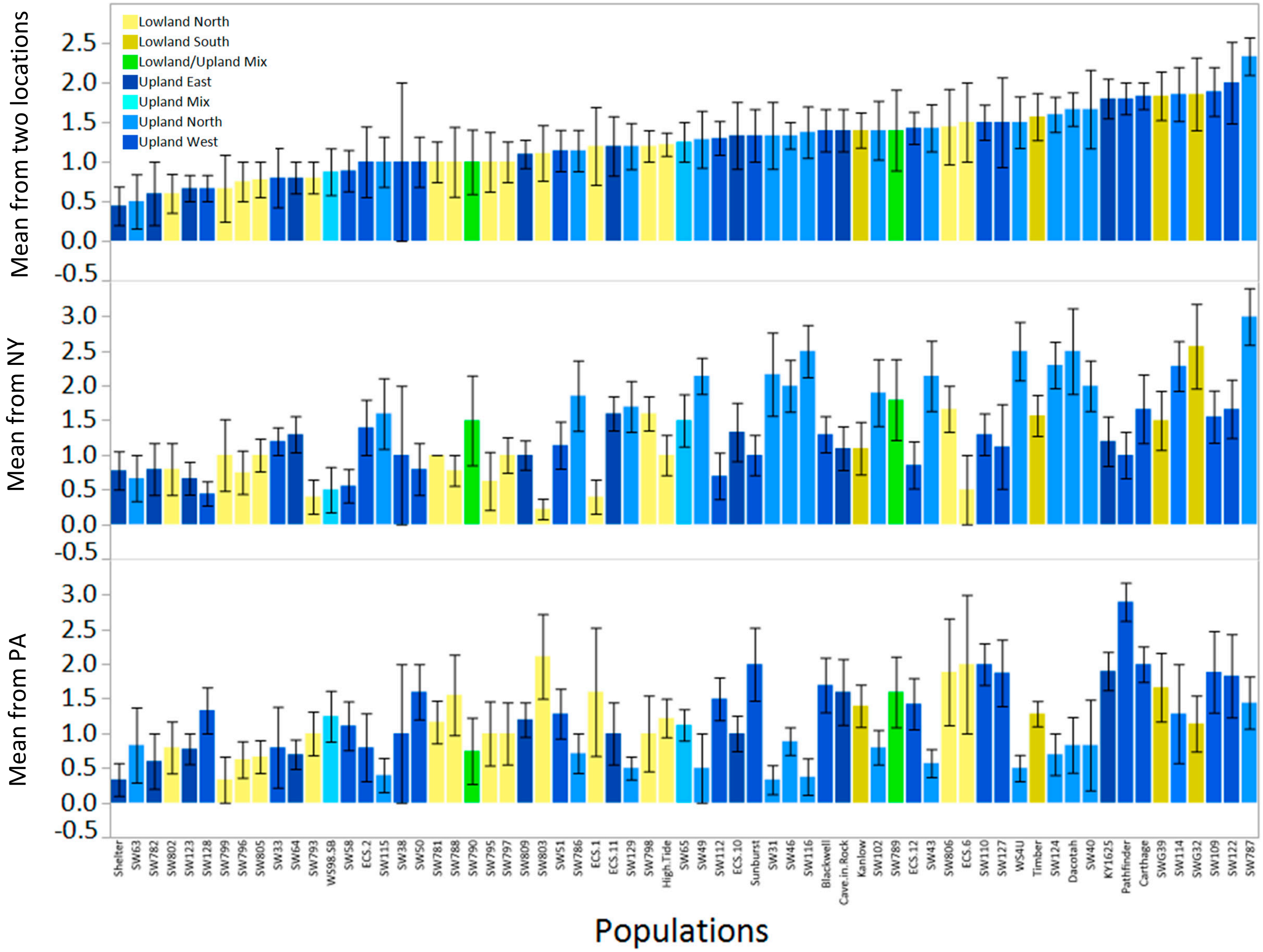

Figure S1. Bar chart of mean severity score from 0 to 5 from two location (top), mean from NY (middle) and mean from PA with standard error from each 66 population. They were ordered based on mean from two locations and colored based on ecotypes.

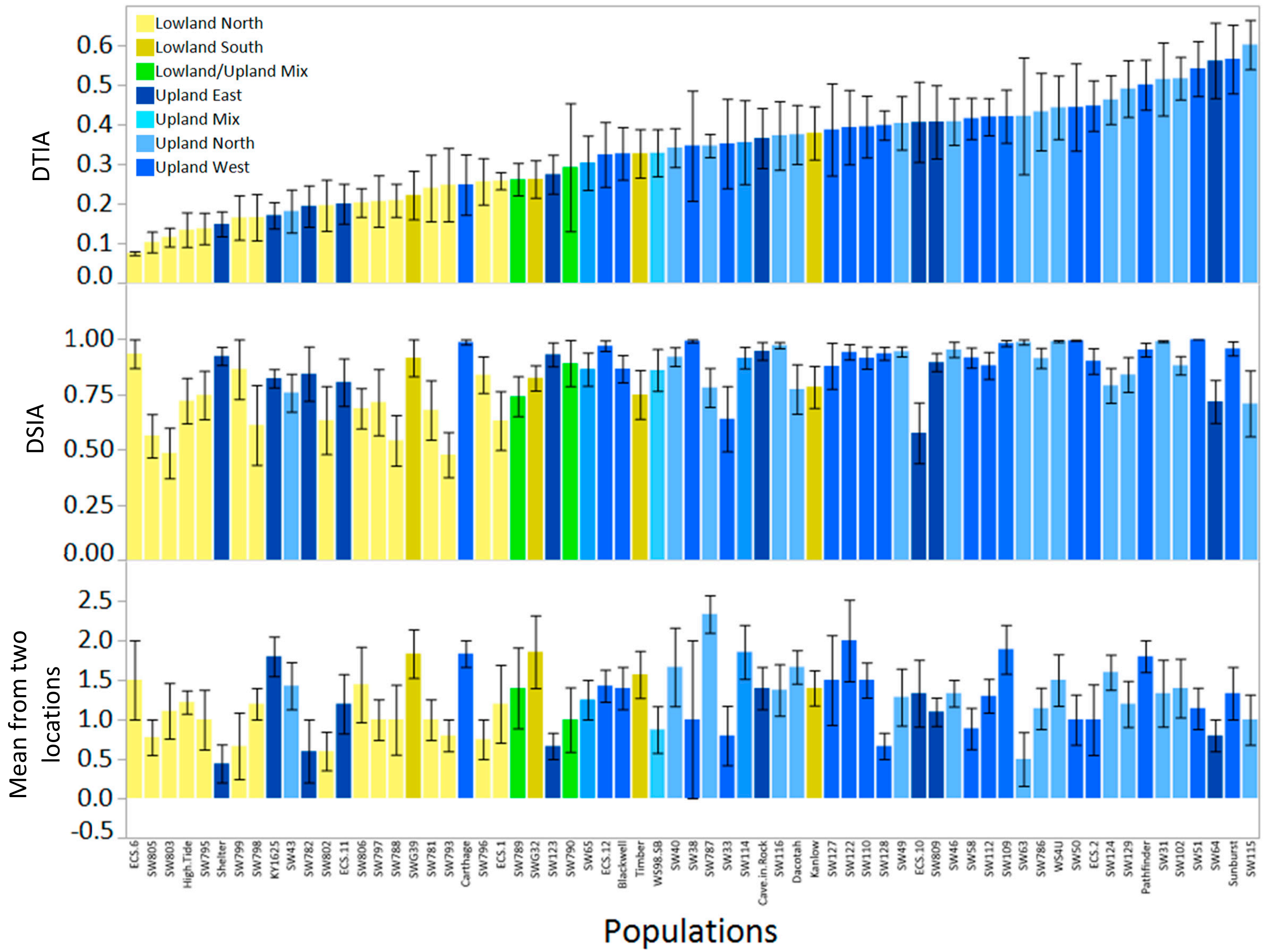

Figure S2 Bar chart of mean severity from the leaf detachment assay via image analysis (DTIA) (top), from the leaf disk assay via image analysis (DSIA) (middle) and mean field severity from two locations with standard error from each of 66 populations. They were ordered based on DTIA and colored based on ecotypes.

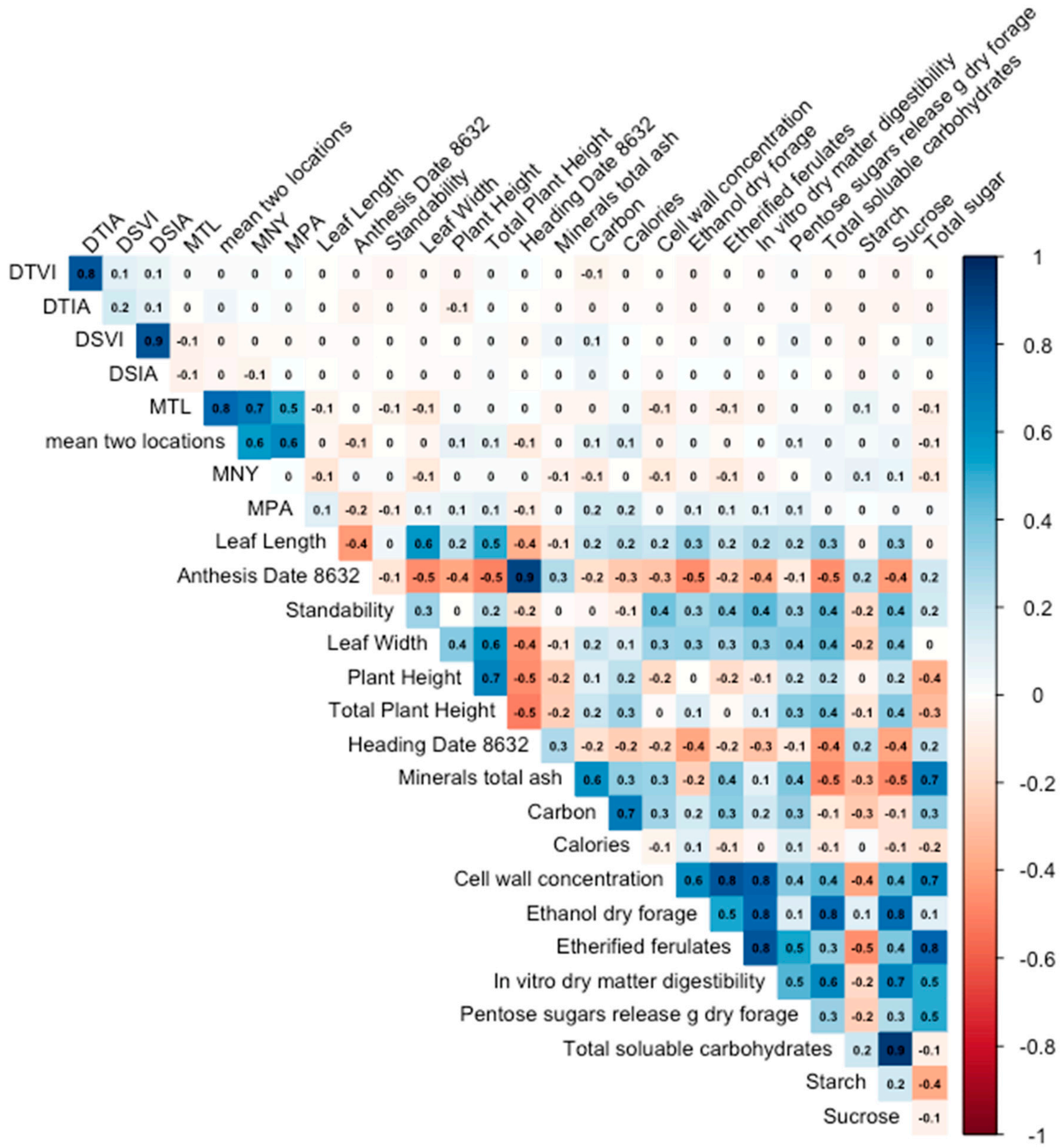

Figure S3. Correlation plot among BLUPs of severity from the leaf detachment via vision (DTVI), via image analysis (DTIA), from leaf disk assay via vision (DSVI), via image analysis (DSIA), the highest score between two locations (MTL), mean from two locations (mean TL), highest score in NY (MNY), highest score in PA (MPA) and BLUPs of other agronomic and biomass quality traits.

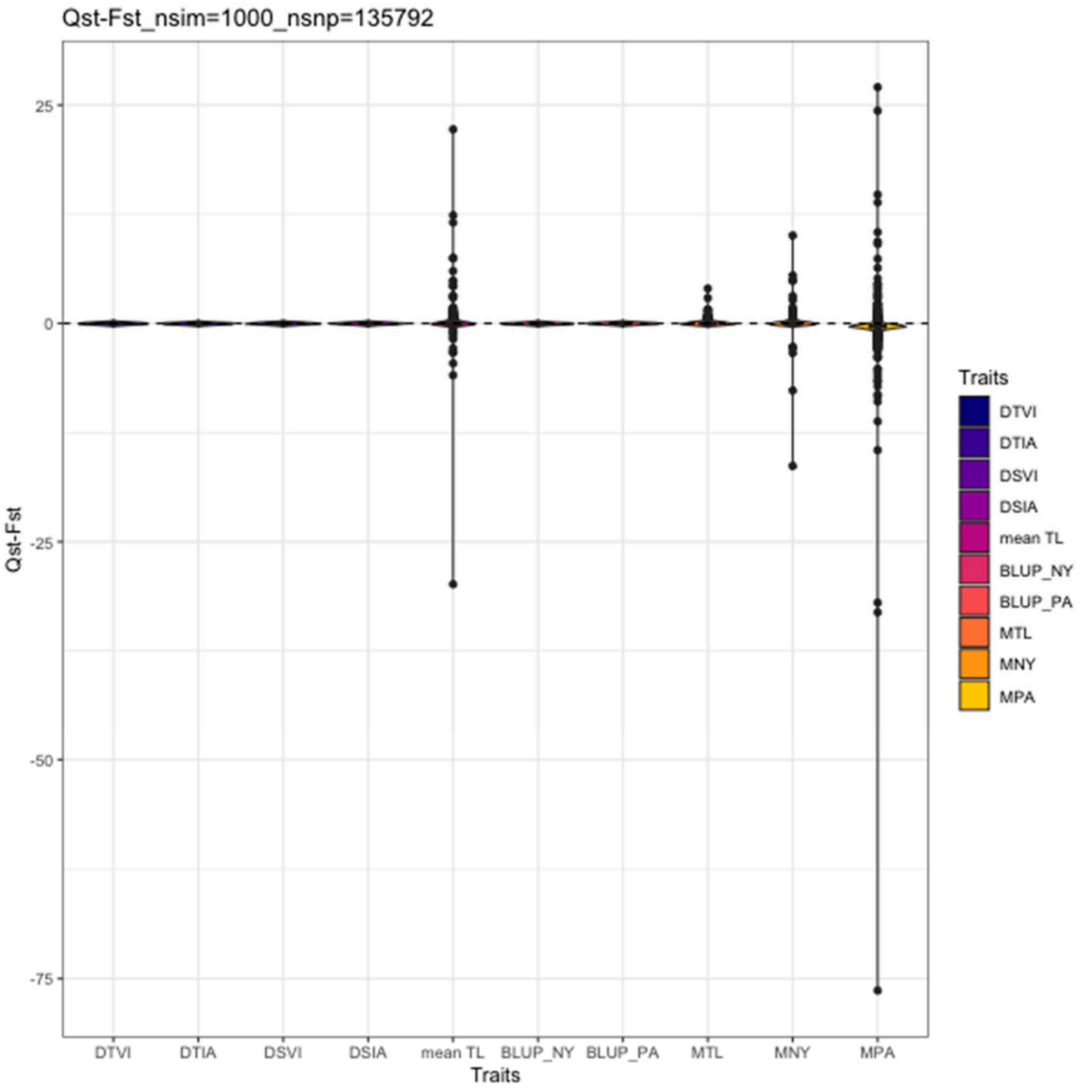

Figure S4. The bootstrapped distributions of Qst-Fst for each phenotypes (DTVI, DTIA, DSVI, DSIA, mean TL, BLUPs NY, BLUP PA, MTL, MNY, and MPA) is compared against the expected value of zero under neutrality (black dashed line).
