# Supplementary material for: Genome-Wide Associations with Resistance to Bipolaris Leaf Spot (Bipolaris oryzae (Breda de Haan) Shoemaker) in a Northern Switchgrass Population (Panicum virgatum L.)
Source: Plants (Basel). 2022 May 20;11(10):1362. doi: 10.3390/plants11101362 (PMC9144872; doi:10.3390/plants11101362)
Supplement: Supplementary file 1 [file plants-11-01362-s001.zip › Songsomboon_supplement_file_Plant_revision.pdf]

Heritability Equations

Severity from the detached leaf (DTVI and DTIA)

$$H_{DT}^2 = \frac{\sigma_g^2}{\sigma_g^2 + \frac{\sigma_e^2}{rep * \exp} + \frac{\sigma_{ge}^2}{exp} + \frac{\sigma_{gp}^2}{plate}}$$

Severity from the leaf disk assay (DSVI and DSIA)

$$H_{DS}^2 = \frac{\sigma_g^2}{\sigma_g^2 + \frac{\sigma_e^2}{rep * plate} + \frac{\sigma_{ge}^2}{plate}}$$

Severity in field from two locations

$$H_{TL}^2 = \frac{\sigma_g^2}{\sigma_g^2 + \sigma_e^2}$$

Severity in field from NY

$$H_{NY}^2 = \frac{\sigma_g^2}{\sigma_g^2 + \sigma_e^2}$$

Severity in field from PA

$$H_{PA}^2 = \frac{\sigma_g^2}{\sigma_g^2 + \sigma_e^2}$$

$\sigma_g^2 = variance\ due\ to\ genetic$

$\sigma_e^2 = variance\ due\ to\ residual\ error$

$\sigma_{ge}^2 = variance\ due\ to\ interaction\ between\ genetics\ and\ environment\ like\ experiment$

$\sigma_{gp}^2 = variance\ due\ to\ interaction\ between\ genetics\ and\ plate\ in\ leaf\ disk\ assay$

$rep = number\ of\ replicates$

$\exp = number\ of\ experiment\ set$
